# Making Sense of Mobile- and Web-Based Wellness Information Technology: Cross-Generational Study

**DOI:** 10.2196/jmir.2124

**Published:** 2013-05-14

**Authors:** Daniel Kutz, Kalpana Shankar, Kay Connelly

**Affiliations:** ^1^School of Library and Information ScienceIndiana UniversityBloomington, INUnited States; ^2^School of Information & Library StudiesUnversity CollegeDublinIreland; ^3^School of Informatics and ComputingIndiana UniversityBloomington, INUnited States

**Keywords:** ubiquitous computing, health promotion, wellness, human-computer interaction, design, generational differences

## Abstract

**Background:**

A recent trend in personal health and wellness management is the development of computerized applications or information and communication technologies (ICTs) that support behavioral change, aid the management of chronic conditions, or help an individual manage their wellness and engage in a healthier lifestyle.

**Objective:**

To understand how individuals across 3 generations (young, middle-aged, and older) think about the design and use of collaborative health and wellness management technologies and what roles these could take in their lives.

**Methods:**

Face-to-face semistructured interviews, paper prototype systems, and video skits were used to assess how individuals from 3 age cohorts (young: 18-25 years; middle-aged: 35-50 years; and older: ≥65 years) conceptualize the role that health and wellness computing could take in their lives.

**Results:**

A total of 21 participants in the 3 age cohorts took part (young: n=7; middle-aged: n=7; and older: n=7). Young adults expected to be able to actively manage the presentation of their health-related information. Middle-aged adults had more nuanced expectations that reflect their engagement with work and other life activities. Older adults questioned the sharing of health information with a larger audience, although they saw the value in 1-way sharing between family members or providing aggregated information.

**Conclusions:**

Our findings inform our suggestions for improving the design of future collaborative health and wellness applications that target specific age groups. We recommend that collaborative ICT health applications targeting young adults should integrate with existing social networking sites, whereas those targeting middle-aged and older adults should support small social networks that rely on intimate personal relationships. Systems that target middle-aged adults should support episodic needs, such as time-sensitive, perhaps intermittent, goal setting. They should also have a low barrier to entry, allowing individuals who do not normally engage with the Internet to participate with the application for the specific purposes of health engagement. Collaborative ICT health applications targeting older adults should allow discreet 1-way sharing, and also support sharing of information in aggregate with others’ data. These systems should also provide mechanisms to preselect recipients of different kinds of data, or to easily direct specific information to individuals in real time.

## Introduction

Information and communication technologies (ICTs) that can harness the knowledge and support of other people and allow individuals to manage and understand their health and wellness can empower individuals to actively manage their health, change their behaviors, and learn more about health conditions [1,2]. Examples include general social networking platforms, such as Facebook [3], online patient communities [4], smartphone applications [5], and exercise-oriented video games [6]. These applications generate data about and for the individual, and data that can influence their health-related decision making and technology adoption. Individuals’ preferences about using such applications, useful features, and related factors will be predicated upon their previous experiences with ICTs, similar systems, and other contextualizing concerns, including what others think about them. The popularity and potential of user-targeted health applications for personal empowerment argues for research that can provide us with a deeper understanding of how people perceive such technologies and their interests and concerns about sharing health-related information.

Many of these commercially available wellness-focused ICTs seem to take a one-size-fits-all approach. However, it stands to reason that generational and other demographic differences influence how people think about health in general, the role of personal relationships in health management, and new technologies. A recent study by the Pew Research Center [7] shows that all generations are using technology more often, but for different purposes. These differences should be examined more closely if ICTs are to be effective in promoting healthy behavior across all generations. Furthermore, generational differences in technology adoption and use of health and wellness management may suggest design implications for understanding the generational differences when it comes to the use of ICTs for health.

### Background and Rationale

In this paper, we examine generational attitudes, interests, and motivations toward the use of ICTs for wellness tracking. Comparative studies on the use of ICTs for health and wellness are generally focused on the role and efficacy of using online sources for obtaining health information online [8-12] or has tended to focus on a particular population (eg, caretakers of older adults [13] or teenage girls [14]), or tailoring solutions to meet immediate health needs (eg, diabetes patients [15], overweight children and adolescents [6], juvenile arthritis [16], and physical activity intervention of young populations [17]). The foci of such studies seem to be on chronic conditions [18-20], take a conceptual approach [21-25], or do not break down generational differences in sufficient detail [26-30].

The body of research on older adults and ICTs also seems to reinforce a design-centered approach of examining preexisting systems and identifying usability issues. For example, Pak et al [31] examined how older and young adults navigate information systems and found results that were consistent with preexisting research, namely that older adults use computing technology less than younger adults and lack spatial and orientation abilities. Haverhals et al [18] examined the use of personal health applications for medication management and identified 5 usage challenges (information seeking, having autonomy in treatment decisions, identifying medication dosages, information discrepancy in identifying therapies, and health-information coordination). Kim et al [32] examined how older adults use a personal health record and found that they do not actively engage with these systems due to low technological skills, health literacy, and cognitive capabilities. Nijland et al [30] examined an Internet-based self-care system and identified design issues (lack of tailoring, navigation, efficiency and reliability issues, and legal concerns) that impede use and understanding.

Although this type of work is valuable, we argue that there is more than usability and the cognitive/physical deficit to focus on in the aging population. Instead, attitudes and concerns need to become part of the research agenda as part of the larger societal shift toward personal responsibility for health and wellness and the use of ICTs for doing so. These trends have been brought on by a variety of factors, including economic necessity, the availability of epidemiological knowledge (ie, awareness of health trends at the population level), and a new “moral regime” that advocates for the existence of healthier societies [33]. With this emphasis on prevention comes an expectation that individuals will actively engage in the management of their health. In parallel, the widespread availability and comparatively low cost of information communication technology has resulted in the emergence of numerous commercial products that allow for the tracking and sharing of health information. For the individual, ICTs provide many options for obtaining health information, tracking exercise and diet, and communicating with medical professionals, and as existing research shows [1,2], these systems promote healthy behavior.

### Objective

The objective of our study was to better understand individuals’ motivations for wanting to use ICTs for wellness tracking (if at all) and the generational differences that exist.

Given the popularity of ICT’s for health and wellness tracking, it is essential that researchers understand generational differences among different age groups as they engage with health and wellness technology. A better understanding of how individuals from various age groups conceptualize health and wellness tracking and sharing will allow for creation of better-tailored ICTs that can promote mediated behavior change.

It is essential that researchers understand generational differences among different age groups as they engage with health and wellness technology. Although there have been studies that investigate these generational differences, they have primarily focused on usability concerns [18,30-32] or research on why older adults do not use health-related Internet resources [9-12] What has received less attention is research on motivation, that is research that goes about obtaining a better understanding of how individuals from various age groups conceptualize health and wellness tracking and sharing. An understanding of these motivations and concerns will allow for creation of better-tailored ICTs that can promote better-mediated behavior change. To date, little work exists that examines generational conceptualizations of health technologies. We intend to address this gap in this paper.

## Methods

Our prototype health-tracking systems were presented to 3 distinct groups of individuals: young adults (aged 18-25 years), middle-aged adults (aged 35-50 years), and older adults (age 65 years and older). We had a total of 21 participants with 7 in each age category (Table 1). Interviews were held in the fall of 2010 and spring/early summer of 2011. The Indiana University institutional review board approved our protocol. After obtaining informed consent, we administered a brief questionnaire to each participant in which we asked basic demographic questions and questions about the participant’s previous experiences with various ICTs and health applications. We showed brief videos that explained the functionality of our health-tracking systems and followed with a 1 to 1.5 hour semistructured interview, accompanied by visualizations related to the tracking system.

### Study Design

We developed 2 hypothetical personal health-monitoring systems that would allow us to probe individuals’ perceptions toward the collection, sharing, and transmission of health-related information (ie, context data about diet and exercise that can be interpreted, shared, or examined). The aim of our project was not to focus on the usability of any particular system, but instead to explore a user’s motivation in order to elicit a broader discussion around the kinds of technologies (location sensing, social networking, information sharing, and collaboration) that are increasingly being used in pervasive health applications. Our approach is a formative approach inspired by work done by Beaudin et al [34] who created conceptual mock-ups in order “to elicit feedback about longitudinal tracking ideas” which serve as “probes to elicit detailed reactions and self-reflection during interviews.” We used a combination of mock low-fidelity prototypes [35,36] and video skits [37-39] to provide background information about the applications and suggested uses for the system. Because we were dealing with a hypothetical health-tracking system, we had to choose interaction metaphors that would push for a broader discussion and provide some similarity, while at the same time making sure it did not go too far off-field where the participants would not understand how the system functions.

The research participants were in different stages of life; therefore, we chose to create video skits that would reflect these differences (campus living versus home living) to help better facilitate envisioning and understanding of the technology. The use of a video skit is informed by Mancini et al [37] who used it as a methodological tool to help the viewer understand the ubiquitous technology presented to participants. The first video system demonstrated the use of a campus-based debit card (referred to as campus card) that university students could use to purchase food, either in campus eateries or in local shops and restaurants. The accompanying video showed a student purchasing food items in a general shop located in a school dormitory. A voiceover narrated the problems involved in maintaining a healthy diet while at school. The narrator then introduced our hypothetical food tracking system as a way to track and manage food choices.

The second video illustrated a hypothetical “smart kitchen surface” on which users could place food items and it would then weigh and track what was consumed. The accompanying video showed a middle-aged adult shopping at a local supermarket and then going home to interact with the smart kitchen. The voiceover again presented the challenges of healthy eating and introduced the prototype system as one tool for managing one’s eating habits.

After participants watched both videos (the order of the videos was randomized), we showed them the 2 sets of visualizations related to the prototypes. Semistructured interview questions first probed participants about what came to mind when they saw the mock-ups; this provided us with insight with respect to what understanding they were bringing to the interview about similar systems. Further questions asked them to provide an explanation of how they expected the system to function, if and what information was being tracked, if and how information was being shared, if they would feel comfortable using such a health-tracking system, and if such a system would be useful to them or to anyone they know. We chose to do semistructured interviews because it allowed us to follow conversational segues, which provided us with a richer set of data. For example, it uncovered religious-cultural and commercialized agricultural-industry concerns that we would not have been able to collect if the interview was fully structured.

The first author analyzed all interview transcripts using inductive thematic analysis to look for emergent themes and patterns without necessarily relying on a preexisting framework or set of ideas [40]. All authors then discussed themes and refined them. Subsequent passes through the data were used to code for intergenerational differences among the themes of interest. The process was repeated until consensus on findings was reached.

**Table 1:** Demographic summary of the participants in the study (N=21).

Group and participant #			Sex	Profession	Technology comfort level	Education
**Young**						
	1		F	Master’s student	Comfortable	Bachelor’s degree
	2		M	Unemployed	Somewhat comfortable	Bachelor’s degree
	3		F	Undergraduate student	Very comfortable	Some college
	4		F	Undergraduate student	Comfortable	Some college
	5		F	Doctoral student	Comfortable	Master’s degree
	6		F	Unemployed	Uncomfortable	Juris Doctor
	7		F	Speech therapist	Somewhat comfortable	Master’s degree
**Middle-aged**						
	8		F	Administrative	Uncomfortable	Bachelor’s degree
	9		F	Administrative	Very comfortable	Master’s degree
	10		F	Doctoral candidate	Somewhat comfortable	Master’s degree
	11		M	Supervisor	Very comfortable	Bachelor’s degree
	12		M	Doctoral candidate	Comfortable	Master’s degree
	13		M	Computer programmer	Very comfortable	Master’s degree
	14		M	Carpenter	Comfortable	High school
**Older**						
	15		M	Retired doctor	Comfortable	MD
	16		M	Retired faculty	Somewhat comfortable	PhD
	17		F	Retired manager	Uncomfortable	Some college
	18		M	Retired detective	Somewhat comfortable	Some college
	19		M	Psychotherapist	Uncomfortable	Master’s degree
	20		F	Retired teacher	Somewhat comfortable	Master’s degree
	21		M	Retired therapist	Uncomfortable	Master’s degree

### Prototype Development Process

**Figure 1:**
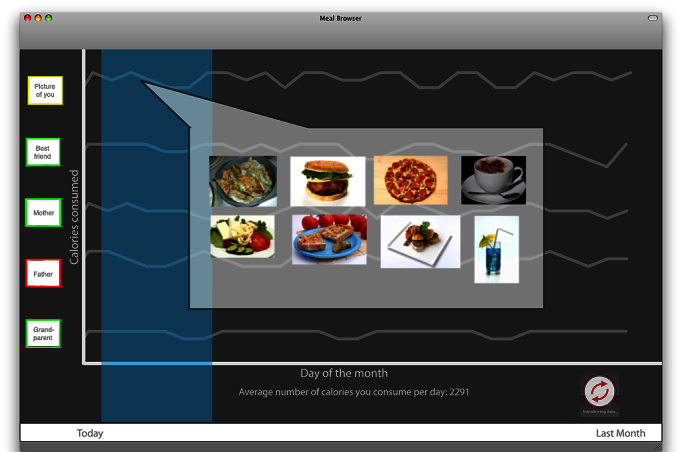
Social networking–inspired mock-up.

**Figure 2:**
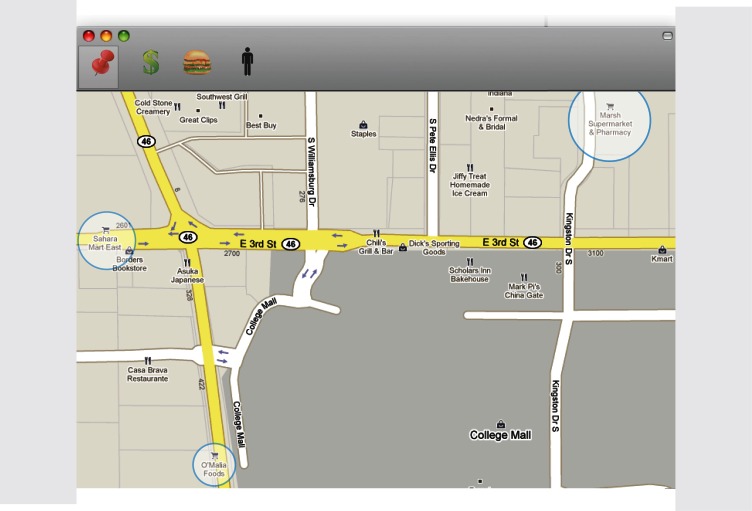
Geographical-inspired mock-up.

We created 2 sets of mock-up interfaces that further explained the functionality of our hypothetical video systems. The first set was explicitly designed to represent mapping systems that are present in smartphones, cars, portable devices, and computers (eg, Google Maps or Microsoft Bing). However, instead of tracking distance to geographic markers, the system tracked individuals’ food purchasing habits as they purchased groceries and meals at restaurants. The intent of the system was to show the user their meal and food purchasing patterns in a geographical fashion. The second series of mock-ups was evocative of a social network–sharing site. This mock-up showed thumbnail profiles of individuals next to what looks like a timeline or historical graph. The goal of this mock-up was to show an interface that was representative of a social networking site; however, in this case nutritional information was being shared. What was being tracked and shared was nutritional health information, what foods were consumed by these individuals, and the nutritional content of the food consumed. We chose these 2 specific metaphors for representing our wellness system because we expected participants to be most familiar with social networking and using online maps. We did not use a simpler or a more common genre of existing information systems (eg, email or an online e-commerce website) because we wanted to use metaphors that could be used to show people’s behavior or geographic information. We wanted to create a generalized prototype that would allow for the exploration of a user’s conceptualization, while at the same time not having it be too limiting (eg, by showing a Facebook page) or so far off-field that the user would not understand what the prototype was that we were showing. Example screenshots of our mock-ups are shown below in Figures 1 and 2.

We created several screenshots of the mock-up for each system, each of which highlighted different features of the prototype’s purpose and functionality. When presented with these mock-ups we asked our participants to verbalize to us their conceptualizations and expectations of each system based on both these interfaces and the accompanying videos. To prevent any potential order bias, we randomized which interface was shown first to the participant.

It is important to recall that our goal was not to evaluate a particular system, but to use the mock-ups to serve as conversational probes in order to elicit participants’ understanding, comfort toward, and perceived usefulness of the data being captured, stored, and shared. Both our pilot and study data indicate the probes served this purpose.

### Changes to the Study

The video for young adults focused on the purchasing of food whereas the video for older adults focused on food preparation. We initially intended to only show the age-oriented video to each group with the middle-aged adults seeing the video created for the older adults. However, when we tested the research protocol, participants raised questions that were addressed in the age-oriented video not shown to their age group. Therefore, we decided to show both videos to all participants during the study. We initially considered that there would be a risk of young adults not being able to associate with the content in the older adult video content and vice versa. However, we decided that this risk was minimal because both videos showed common activities (the purchasing of food) that all age groups engage in.

### Participants

#### Recruitment

Members were recruited via flyers distributed at various locations, including the university health center, university speech and hearing clinic, local retirement communities, libraries, and other local institutions.

#### Technology Experience

Participants answered a brief questionnaire about basic demographics (education, age, and gender) as well as previous use and ownership of and general familiarity with ICTs. Young adults self-reported as the most technologically knowledgeable demographic, with most stating in the survey that they were comfortable to very comfortable with the role that technology played in their lives. Middle-aged adults were mixed in their responses. Older adults primarily reported that they felt uncomfortable around technology. Younger adults primarily used laptop computers and owned cellphones. They also all responded that they used some form of social networking software. These findings are not surprising, as existing survey research by the Pew Research Center indicates that 83% of Millennials (young adults aged between 18-33 years) interviewed use social networking sites [7]. Middle-aged adults’ computer usage was split between desktops and laptops with cellphone ownership being unanimous. Older adults were more likely to own a desktop computer instead of a laptop. These findings match results from a study by Pew Research Center that indicated that 70% of Millennials owned a laptop, compared to 43% and 33% ownership by the older Boomer generation (adults aged between 57-65 years) and the Silent generation (adults aged between 66-74 years), respectively [41].

## Results

In this section, we discuss the 3 identified themes that displayed clear generational distinctions that coalesced from the interview data: passive and active engagement, data and information sharing, and social networking. An overview of our findings is presented in Table 2, and a more detailed discussion of our identified themes follows.

### Data and Information Sharing

Our prototyped systems drew upon existing research that showed that a person is better able to engage in positive health behaviors when they have the social support of an existing network of peers [42,43]. However, analysis of the interview transcripts suggested distinct differences in how the age groups felt about using a mapping or social networking application to share one’s health-related activities. The young adults tended to see significant value in sharing information because it would allow for friends to provide support. These individuals expected that if they posted something, it was open to comment and that was a welcome form of support: “Somebody could say, ‘Wow, you really should watch your salt, or your sodium intake,’” and “‘Looks like you’ve been taking in a lot of vitamin B_12_ this week...that could be dangerous for this organ,’” and “You’re wagging your finger at them, ‘You’re not being so healthy this week, I saw what you bought’” (Y2). Others brought up the ability to show off or brag when doing well as a way to motivate positive behaviors in others: “[It] might be the case that you would need to share that information, to either validate what you are saying, or just brag about it even,” and “We are all able to view these to kind of motivate each other” (Y3).

Middle-aged adults integrated the potential use of these health and wellness tracking systems into the framework of their existing social relationships. For this group, the broadcasting of activities was also seen as being useful in helping support more intimate social relationships for specific health goals, as opposed to the younger group’s willingness to sharing data more broadly. For example, M9 said, “I always have a workout partner with me that knows this is what my goal is...and if I start slacking, then I want you to say, ‘Are you feeling okay?’” and “I really can’t see why I’d want to know what a particular person consumed, unless I was perhaps a parent looking at the student.”

Older adults could see the benefit in sharing health data with their family and close friends. The focus on family by older participants is in-line with previous research that showed that older adults often feel they have a moral duty to see and provide suggestions about health to their children [44]. In other scenarios, older adults frequently expressed that sharing did not make sense to them. They often just didn’t see why someone outside of the family might be interested: “...don’t know why somebody would care about this other than my family” (O17). Similarly, they did not see why they would care to see anyone else’s information. When questioned if there would be any value in viewing the shopping and dietary habits of other individuals, O17 responded with a clear no: “I can’t think of any reason...unless you’re just plain nosy!”

When asked who should be able to see their information, O16 responded, “...any researcher can see this or that you can see it because I want you to monitor my diet or I’m interested in your research program.” However, when asked if in return they would be able to see the dietary habits of the researcher they responded with: “I don’t care what you eat. I would object to a system that allowed me to look at anybody that I wanted to see what they were eating.”

### Social Networking

Another clear distinction identified across generations was the familiarity with and expectations from social networking software. All members of the youngest age group quickly identified the social networking mock-up, and to a lesser extent the map mock-up, as having a very strong social networking component to them. Once this group identified the social networking connection, the mock-ups immediately made more sense. They described information sharing using metaphors drawn from social networking technologies (eg, lists and profiles): “Sharing would be to people, maybe that you’ve already selected” (Y1). They discussed the implicit expectation of reciprocal sharing of information: “...other people in my family or my household, or friends that I could maybe do some social networking with, and see, ‘Oh, what’s Sarah been eating this week?’” (Y2). They also clearly felt they could control information flow about themselves through managing lists of friends: “I have different groups of friends, so maybe I have a family group, but maybe I also have a work group. And so maybe I could select who I want to share the info with” (Y3). This suggests the active control and expectation that this age has when it comes to managing their online personae and what is shared with others. Younger adults also referenced sharing even when social networking was not explicitly embedded in the interface. For example, when asked about the functionality of the map health and wellness tracking system, Y3 responded: “...you can add friends on kind of a like a GPS [Global Positioning System] and then if they are logged into certain places, then you can say, we are both at [a local grocery store] and maybe meet up.”

Four of the participants in the middle-aged group (M9, M11, M12, and M14) referred to social networking, but with less frequency and detail than the younger adults. The youngest middle-aged adult, 35-year-old M13, made a strong association with social networking. The other 2 individuals in this group (M8, M10) did not make associations with social networking until much later in their interviews. For example, in the case of M8, it was only after the interviewer highlighted and explained some of the features and functionality of the health tracking did the participant draw parallels to social networking sites: “I mean now I’m starting to try and compare it to something like Facebook.”

Additionally, the middle-aged adults who referred to social networking indicated that these systems were perceived as a diversion...something to do with free time: “It could just be fun to check some things out, similar to a Facebook-type interaction” (M11). They were also aware that they approached social networking differently than their younger counterparts: “There is definitely a generational split. The younger individuals, they see this information different [*sic*]. They have different perceptions of privacy of information” (M9). Participant M10 found it strange that people would constantly update their Facebook profile and indicated that he was more likely to curate: “I judiciously determine that something would be of interest to a large number of people.”

The older adults’ expectations of how the health and wellness tracking systems functioned were far more varied. Participant responses tended to be informed by the technologies they had encountered in their careers, confirming findings in previous research [45]. Participant O15, who had a medical background, referred to the thumbnails of individuals and activity data on the social networking modeled health and wellness tracking system as being like a cardiogram or medical record. He did not see the purpose of viewing an individual’s health behavior; they considered the data to be representative of an entire population: “General population. I would think that this represented. If we multiplied this by 100,000 people, I would guess this would show some descriptive statistic, median or mean.” A former professor, O16 frequently referenced databases: “It would be putting data into a database that kept a running track of what I was doing, eating, whatever. It would be updating essentially.” However, databases were not the only analogy employed during the sense-making process by the older adults. Participant O16 hypothesized several ways in which data could be collected or retrieved, from natural language queries (“I could simply ask a question”) to manual data entry.

### Emergent Theme: Passive and Active Engagement

One of the strongest and unexpected age-related themes we identified is that participants would either respond in what we call a passive or active manner when discussing how they would expect to engage with a health-monitoring system. Unlike the social networking theme, which is something we were specifically probing for, the passive and active engagement themes are an emergent finding. An individual taking a passive approach would expect the technology to do what is required with minimal intervention by the user (if they had a positive stance toward the system), or if they had some concerns, participants would express the expectation that nothing could really be done outside of opting out. However, individuals that expected to take a more active approach with their technology use expected the system to provide them with the right resources and capabilities to do so. This group expected these tools would allow them to adjust how the system functioned, curate data, and control data sharing and broadcasting.

Older adults were more likely to take a more passive approach and be willing to delegate responsibility to other actors to managing access to the data and the system itself. For example, when asked from whom information should be hidden, O16 stated: “That’s a good question to be determined by the people who set up the system.” Other older respondents were willing to delegate responsibility to the system itself, which suggests that that the respondent trusted the system to be capable of correctly evaluating and managing their health-related information. For example, when asked who would have access to dietary information, O17 stated: “That would be a decision for the system to make.” She later said: “I trust it.”

Although the responses from middle-aged adults were mixed, what makes this group stand out from the other 2 is their focus on and concerns for the effects that collaborative health-tracking technology would have on their jobs and careers. For example, M9 said she would consider using a health-tracking system on a limited basis with “real life” friends, but her main concern was that her employer not have any access to this information: “If...[employer] is not going to be able to scan our little card and track what we’re doing and see what we’re doing, I would have no problem using a program like that.”

The young adult participants consistently used active language when describing the systems. They expected to be given tools that would allow them to actively manage their online persona via lists, access controls, and updates. They expected that they would have control of their data, or at least how their data would be made available to the public or certain individuals. When asked if they would be willing to share their data to support healthy behavior, Y3 replied: “Yeah, and I think I would be...as long as I had, you know, the privilege, I guess, to be able to modify that whenever I wanted to, and select if I wanted to share how and whenever I wanted to ...if I’m choosing to be a part of this type of program and I was able to just do the select the people that I wanted, or whatever, then I would imagine that I would share that information. Otherwise, I wouldn’t be a part of the program.”

Based on our identified themes, we believe that the generational differences we elicited in this study have significant generalizable design implications for designing new health and wellness technologies.

### Interpretation of Themes

Although we discussed 3 themes as distinct findings for the sake of clarity, they were strongly interrelated. For example, when discussing the process of broadcasting health-related information subject, Y3 stated: “...show it with this predetermined group that I had already selected, then it would be more motivating than just showing it to myself.” This response illustrates the preferred metaphor of this age group (social networking) expressed via the concept of predetermined groups. However, it also expresses a sense of active engagement with controlling the system (“that I had already selected”) and the assumption that sharing information about one’s health activities would be more motivating to engage in healthy behavior.

As we described, young and some middle-aged adults drew upon metaphors from social networking software to explain how these systems would work and the meaning it would have in their lives. For the younger generations, social networking tools are essential adjuncts to their lives. As such, it is not surprising that they would draw parallels to social networking technology in our health-tracking mock-ups. However, for middle-aged (and older) adults, online social networks were primarily seen as a novel distraction to supplement preexisting established social networks and as a way to occasionally check-in on contacts. Older adults’ reactions to these systems, however, were frequently ones of cautious skepticism. This is consistent with previous research that shows older adults will respond with ambivalence to computing technology that, in their mind, does not address their day-to-day needs [46].

Their strong connection to social networking may explain why young adults also took an active approach toward managing their online persona, whereas older adults, who gravitated toward databases and similar work-oriented applications (over which they would presumably have less personal or individual control) to describe the system, did not. Younger adults also took an active approach toward managing other people’s perceptions of them. For this generation, manipulating and crafting how one is represented online is a perfectly reasonable strategy to employ: “Because it sounds kind of like things you can almost like play around with, because it gives you option to manipulate data” (Y4). Older adults that spoke passively were uncomfortable of such management: “Oh hell, there is no room for lying. I wouldn’t share that. Not at all, I mean, if you are lying, then I think that negates everything” (O16). Participant O19 considered this online management as not being truthful: “People just mostly tell all the shiny, happy side of themselves and nobody really knows what’s going on by looking at that stuff.”

The young adults stated that they would publicize to groups or lists, which again indicates the use of social networking software metaphors and an active management approach. However, the middle-aged adults stated that they would possibly publicize their information, but only for an audience of existing friends with which they are actively and collectively striving toward certain health-related goals. The older adults did not see any use in publicizing health-related information, and only after further discussion would they acknowledge that possibly there could be a value in broadcasting, but only to other family members. The 2 older groups raised concerns about broadcasting health-related information to potentially inappropriate audiences, such as employers.

For older adults, the concept of sharing is not related to broadcasting, but sharing information for research purposes or the family unit. These differences reinforce findings from the aging literature that have found that the quality of relationships is preferred over quantity as people age so information tends to be shared with fewer people, but more deeply [47]. A system that is designed solely around the idea of sharing health-related information for motivational purposes might not gain much traction among older adults. Designers may need to take this into account when designing for these populations.

**Table 2:** Summary of identified generational distinctions.

Theme	Young adults	Middle-aged adults	Older adults
Participation and engagement	Active engagement	In between	Passive engagement
Data and information sharing	Open sharing with expected response	Sharing of information with existing networks	Sharing does not make sense
Social networking	Tied strongly to sharing and their conceptualization of how systems should function	Acknowledgement of awareness, although tied to existing networks	Some awareness, but not direct interest in participation

## Discussion

The narratives that people use to make sense of new technologies may vary widely by age, life experience, and concerns external to the ICT. However, we believe that those divergent framings are a potentially rich resource of guiding principles for designers of new health and wellness applications, particularly those targeting specific age groups.

### Design Recommendations

It may be obvious that one would not design the same approach for a 20-year-old and an 80-year-old with respect to other ICTs, but application developers often adopt such one-size-fits-all mindsets toward health and wellness systems.

We present design recommendations informed by the findings of our study. Table 3 provides an overview of these recommendations. These recommendations are meant to improve acceptance and adoption of future health and wellness information systems.

### Social Networking

Our study reflects the way other researchers have found that young adults use social networking sites: although they might desire the ability to broadcast their health information to a large social network for support and motivation, our findings seem to indicate that they expect these applications to afford them the ability to manage content and recipients.

Active management implicitly brings up the question of informational privacy. Although at one time there was the expectation that this age group was somewhat indifferent to privacy, more recent research indicates otherwise [48,49]. These more recent findings are consistent with the results from our study. Young adults expressed that they would want to manage their privacy by drawing upon metaphors of current popular social networking software (lists, groups, and other privacy settings). Our findings suggest that this age group may expect systems to provide them with the tools to actively manage and control their online personae.

#### Design Recommendation 1

Collaborative ICT health applications targeting young adults should integrate with existing social networking sites so users can make use of their current social networks as well as familiar tools for information and network management.

Middle-aged and older adults had more nuanced expectations when it came to sharing health-related information. Middle-age adults expected sharing to be limited to individuals who are part of their real world social networks or subgroups that have a shared interest (eg, fitness groups), whereas older adults envisioned the system to work with familial networks. Existing social networking sites, such as Facebook, encourage larger networks in several ways. Their open nature allows people to search for acquaintances, and allows “friends of friends” to see and interact with each other through a mutual acquaintance. Varying expectations and social conventions lead some people to make friend requests to others who may deem the friendship remote at best. Yet it can be uncomfortable for a person to deny a friend request from a remote acquaintance, or remove someone from their network whom they no longer feel close to. This results in networks that grow over time to be quite large. Thus, although integration of health applications into existing social networks may be inappropriate for many users, the ability to use familiar tools and integrate information and networks across platforms may be a preferred feature.

#### Design Recommendation 2

Collaborative ICT health applications targeting middle-aged and older adults should support small social networks that rely on intimate personal relationships.

This recommendation also addresses the more passive engagement style of older adults as a side effect. With a smaller network, there is less need to actively manage sharing of information through lists and other access control. However, a small network does not necessarily mean that it is desirable to share all information with everyone at all times. Thus, an application should support other modes of sharing than simple broadcast, which is standard fare for current social networking applications.

#### Design Recommendation 3

Collaborative ICT health applications targeting older adults should incorporate mechanisms to preselect recipients of different kinds of data, or to easily direct specific information to individuals in real time.

Fortunately, this is more easily done with a smaller social network. For example, when an older adult links a new pedometer to their tracking software, the interface can show them (on 1 screen) all of the people in their current network and the older adult can select who should receive their step counts and in what form (eg, daily, weekly, or in aggregate). Similarly, if an older adult inputs a new exercise goal, they can specify who should receive that goal from their network. This is a much easier process than when the network is large and a person must decide who among hundreds of friends should have access.

**Table 3:** Overview of design recommendations.

Age group	Recommendation
Young adults	Have preexisting expectations based on social networking experience. Expect integration with existing social networking sites.
Middle-aged adults	Should support smaller social networks and existing relationships. Needs to support different modes of sharing besides broadcast. Have a low barrier of entry. Support intermittent, episodic use.
Older adults	Support different modes of sharing besides broadcast; simplify the process of preselecting recipients to receive information. Have a low barrier of entry. Allow for 1-way or aggregate sharing.

#### Design Recommendation 4

Collaborative ICT health applications targeting middle-aged and older adults should have a low barrier to entry, allowing individuals who do not normally engage with the Internet to participate with the application for the specific purposes of health engagement.

Individuals in these physical social networks may not have a regular online presence; therefore, health-tracking systems should have a low barrier of entry. Examples of how to achieve this low barrier may be to develop a mobile application capable of working on most cell phones, or a low-cost, special-purpose display that can be purchased at a local department store and simply plugged in.

### Episodic Support

Comments from the middle-aged adults indicate that this age group might take a more episodic or goal-oriented approach toward using health information–management systems (eg, to meet a specific goal, such as walking 3 times a week). For example, M12 stated, “I want to be able to start out running and get up to 3.5 miles a day under 30 minutes. That’s my goal. That’s my goal every winter.” As such, a system targeting this age group should facilitate these episodic needs.

#### Design Recommendation 5

Collaborative ICT health applications targeting middle-age adults should support episodic needs, such as time-sensitive, perhaps intermittent, goal setting.

### One-Way and Aggregated Sharing

Some middle-aged adults mentioned the benefit of 1-sided sharing of health-related information, such as a parent wanting to see what their young adult children were doing, but not vice versa: “If you had children and you wanted to watch what they were eating...you could monitor, in a good way hopefully, their eating habits” (M10). A few of the older adults saw value in comparing their health information with aggregated information from a group. For example, O15 considered that the sharing of aggregate information as “being useful in a research study. For example, here is individual variation among adult males or males over 50 or whatever.” This individual did express some concern in regards to anonymity and security: “I would want some reassurance before I got involved in the system, yeah. Just how the data is going to be used.”

#### Design Recommendation 6

Collaborative ICT health applications targeting middle-aged or older adults should allow discreet 1-way sharing and also support sharing of information in aggregate with others’ data.

For example, an older adult may be willing to share their data with others if it appears in aggregate with a larger group, but not on an individual basis. If data presented in the health application interface always appears in aggregate, then the lack of individual data does not indicate a lack of willingness to share with others.

### Conclusion

The recent popularity and availability of computerized wellness and health tracking and sharing systems made us question how users understand and conceptualize such systems. Although the research literature focuses on the use of these systems by specific subpopulations, commercial products seem to take a one-size-fits-all approach. However, health and wellness management is strongly situated at the family level and is a cross-generational activity. As such, it is important to understand cross-generational differences and attitudes toward these health and wellness tracking systems. Our study set out to identify these differences, of which we found several salient examples. These include nuanced expectations of middle-aged adults, young adults’ expectations of being able to actively manage their health and wellness information, and older adults’ interest in smaller social networks and intimate relationships. These expectations need to be taken into account by designers of health and wellness applications if they want to develop systems that target specific generations or be used successfully to support health and wellness across the life span.

### Unanswered Questions and Future Research

We touched briefly upon the privacy and security concerns respondents had with respect to employers, family members, and others accessing their health data, but did not explore these themes thoroughly and will likely do so in the future. Many of these issues in health information have been extensively explored by other researchers as well [50-54]. We are also aware that there is a gender difference across our populations, which may influence technology interest and adoption as well as attitudes toward personal health.

### Limitations

Although the interviews provided us with a rich set of data, we acknowledge limitations. The small size of the group allows only preliminary themes of interest to surface. The individuals we interviewed were similar in socioeconomic status, experience with information technology, and education levels. There are also some limitations in defining our older adult’s category as including all participants over 65 years. In the Pew Research Center’s Internet study, there were some remarkable differences in technology use between the Silent generation (ages 66-74 years) and the GI generation (ages 75+). For example, 58% of the Silent generation were online versus 30% of the GI generation [7]. Lastly, we are also aware that our study has some socioeconomic limitations. Except for 1 individual, everyone had (or was in the process of getting) an undergraduate college education. Furthermore, their socioeconomic status puts all participants into the middle class; thus, we do not have any knowledge how responses would have varied from lower or higher income individuals. Future studies will examine these identified limitations and the impact they have on the use of collaborative health and wellness management technologies.

## Multimedia Appendix 1

This video demonstrates the use of a campus-based debit card (referred to as campus card) that university students could use to purchase food, either in campus eateries or in local shops and restaurants.

## Multimedia Appendix 2

This video illustrates a hypothetical “smart kitchen surface” that would allow users to place food items from their kitchen on a flat surface that would then weigh and track what was consumed.

## Abbreviations

ICT: information and communication technology
